# Specific Capture and Whole-Genome Sequencing of Viruses from Clinical Samples

**DOI:** 10.1371/journal.pone.0027805

**Published:** 2011-11-18

**Authors:** Daniel P. Depledge, Anne L. Palser, Simon J. Watson, Imogen Yi-Chun Lai, Eleanor R. Gray, Paul Grant, Ravinder K. Kanda, Emily Leproust, Paul Kellam, Judith Breuer

**Affiliations:** 1 Division of Infection and Immunity, University College London, London, United Kingdom; 2 Pathogen Genetics, Wellcome Trust Sanger Institute, Wellcome Trust Genome Campus, Hinxton, Cambridgeshire, United Kingdom; 3 Department of Virology, University College London Hospitals NHS Trust, London, United Kingdom; 4 Agilent Technologies, Santa Rosa, California, United States of America; Duke University School of Medicine, United States of America

## Abstract

Whole genome sequencing of viruses directly from clinical samples is integral for understanding the genetics of host-virus interactions. Here, we report the use of sample sparing target enrichment (by hybridisation) for viral nucleic acid separation and deep-sequencing of herpesvirus genomes directly from a range of clinical samples including saliva, blood, virus vesicles, cerebrospinal fluid, and tumour cell lines. We demonstrate the effectiveness of the method by deep-sequencing 13 highly cell-associated human herpesvirus genomes and generating full length genome alignments at high read depth. Moreover, we show the specificity of the method enables the study of viral population structures and their diversity within a range of clinical samples types.

## Introduction

Whole genome sequencing of viral genomes directly from clinical samples is critically important for identifying genetic variants which cause disease, including those that are under positive selection pressure through interaction with the host [1]. Genetic variation defines virus population structures and is used effectively in determining transmission chains [2]. In clinical samples, viral genome copies per millilitre can number in the billions yet the relative proportion of viral nucleic acid is minute in comparison to host nucleic acid. Direct sequencing of mixed human and viral nucleic acids yields representative proportions of sequence reads that map to viral genomes [3], This represents a significant issue when dealing with samples that contain low proportions of viral nucleic acid and one that has limited such studies from being carried out previously [4], [5], [6], [7]. For this reason, current methods for viral genome sequencing benefit significantly from isolation of viral nucleic acid from host nucleic acid prior to sequencing. The primary methods rely on the production of microgram quantities of viral nucleic acid by either *in vitro* virus culture or amplification of virus genomes by PCR [4], [5], [6], [7]. However, both methods are known to alter virus population structures either by replication advantages of subsets of viruses during *in vitro* culture or through the introduction of nucleotide mutations, gene deletions and genome rearrangements [8], [9]. Moreover, the presence of PCR-inhibitory secondary structure and the inability of many viral species to thrive in culture present additional difficulties in generating sufficient quantities of viral nucleic acid for whole genome sequencing. These factors all impact on the accuracy of assembled genome sequences and the interpretation of minority population structures.

Some of the hardest viral genomes to sequence are those of the herpesviridae, a family of large highly cell associated (120–230 kilo base pairs (kbp)) DNA viruses. The eight known human-infective Herpesviruses are currently represented by 29 whole genome sequences in GenBank. 18 of these represent Varicella-Zoster Virus (VZV) strains, the causative agent chickenpox and shingles while Epstein-Barr and Kaposi's sarcoma-associated herpes viruses (EBV and KSHV, respectively) are represented by only two strains each. In all cases, these genomes were sequenced using viral nucleic acid isolated from cultured material [7], [10], [11], [12], [13], [14], [15], [16], [17], [18]).

Target isolation by hybridisation and subsequent enrichment has proven highly effective in exome sequencing studies [19], enabling researchers to target and deep-sequence specific regions within the human genome. This method uses overlapping 120-mer biotinylated RNA baits, designed by tiling across targeted genomic regions. Subsequent hybridisation of the RNA baits with sequence library-prepared nucleic acid enables isolation and enrichment of target material (using a minimal number of rounds of PCR) and generating sufficient quantities for sequencing on second-generation platforms (Illumina, Roche, Abi). Moreover, while microgram quantities of nucleic acid are still required for sequence library preparation, the target genomes need only comprise a fraction of the total nucleic acid [20].

We describe here, the use of a solution-based target capture methodology to separate and enrich for specific viral genomes from low volume clinical samples comprising complex nucleic acid mixtures (including excess human and bacterial nucleic acids). We use a variety of approaches to determine the optimal method for generating sufficient total nucleic acid for sequence library preparation including whole genome amplification methods and the use of carrier nucleic acid. The utility of the method is demonstrated by directly sequencing 13 human herpesvirus genomes from a range of clinical samples including blood, saliva, vesicle fluid, cerebrospinal fluid and tumour cell lines.

## Results and Discussion

Initially, total DNA was extracted from a range of VZV, EBV and KSHV clinical and cultured samples (Table 1 and Table S1 online) and their viral loads determined. Due to the decreased sensitivity of the qPCR assay (versus the PCR assay used to confirm presence of viral DNA), no viral load data could be determined for six VZV samples which were below the limit of detection. Five samples underwent whole genome amplification (WGA) using the high fidelity Phi29 DNA polymerase and random primers to generate sufficient DNA for the library preparation step [21]. Viral load assays, post-WGA, showed a large increase in viral nucleic acid within the samples (Table S1). All remaining samples were prepared without WGA, either directly (all culture samples and clinical sample Vesicle I) or with the addition of carrier DNA (clinical samples Blood I). Sequence library preparation, hybridisation and subsequent enrichment were carried out on all samples using the SureSelect Target Enrichment System (Agilent Technologies) [20] and custom designed RNA baits. For comparison, two cultured samples were amplified by overlapping long PCR and the products mixed in equimolar ratios prior to sequence library preparation. The viral load and human DNA content was determined for each sample at the pre-hybridisation, post-hybridisation and post-amplification stages and are expressed as a ratio (Table 1).

**Table 1:** Deep sequencing of clinical samples prepared using the SureSelect Target Enrichment System.

	Sample	Starting	Sample type	Manipulation	Ratio of Viral DNA: Human DNA			% Paired-end reads mapped	%Genome	coverage	Mean read
		material			Pre-hybridisation	Post-hybridisation	Post-amplification		>5-fold	>100-fold	Depth per base
**VZV**	Culture I	3 µg	Zoster Vaccine Rash	low passage culture	nd	nd	nd	78.66	99.81	98.27	1672
	Culture II	3 µg	Zoster Vaccine Rash	low passage culture	nd	nd	nd	93.98	99.85	98.85	2720
	CSF I	3 µg	Encephalitis	WGA	nd	nd	nd	34.87	99.94	98.28	729
	Vesicle IV	3 µg	Zoster Vaccine Rash	WGA	10299	1157666	9713604	93.69	99.30	97.54	3022
	Saliva I	3 µg	Wild-type Zoster	WGA	2	14	nd	40.15	99.19	94.72	950
	Vesicle III	3 µg	Zoster Vaccine Rash	WGA	34976	1006398	3931100	60.47	99.83	97.88	2416
	Vesicle II	3 µg	Zoster Vaccine Rash	WGA	519875	9855143	856279	96.01	100.00	98.84	1096
	Blood I	250ng*	Wild-type Zoster	none	2	nd	105545	71.14	99.82	97.51	1819
	Vesicle I	500ng	Wild-type Varicella Rash	none	1097	38	nd	99.48	99.93	99.27	3197
**EBV**	JSC1	2 µg	PEL cell line reactivated virus	culture supernatant	nd	nd	nd	69.10	99.34	98.56	2523
	HBL6	2 µg	PEL cell line reactivated virus	culture supernatant	nd	nd	nd	52.84	98.25	97.17	2599
**KSHV**	JSC1	4 µg	PEL cell line reactivated virus	culture supernatant	nd	nd	nd	92.01	99.73	95.47	2471
	HBL6	5 µg	PEL cell line reactivated virus	culture supernatant	nd	nd	nd	90.97	98.19	93.92	1773

nd – not determined due to insufficient sample available |

*2750ng carrier DNA added.

All samples were multiplexed (2-7 per lane) and sequenced using a Genome Analyser IIx (Illumina, Inc) yielding between either 4.8×10^7^–7.2×10^7^ 76bp paired-end reads per sample (clinical and cultured samples) or 2.7×10^7^–3.3×10^7^ 54 bp paired-end reads (long PCR amplicons). Post-sequencing, read-pair quality control was performed using QUASR (http://sourceforge.net/projects/quasr/), and removing duplicate and low quality read-pairs. Consensus genome sequences were produced by aligning read-pairs against a reference genome using the Burrows-Wheeler Aligner [22] while polymorphic loci (including SNPs) were reported using VarScan [23]. The accuracy of SNPs identified in the assembled consensus sequences for culture samples I and II and clinical samples Vesicle II and CSF I was confirmed by either direct PCR and sanger sequencing from the original material or prior reporting of the SNP in peer-reviewed publications [24], [25] (Table S2). In agreement with previous studies, there was no evidence of error-induced substitutions or indels in the consensus sequences of samples prepared using the Phi29 DNA polymerase for WGA [26].

**Figure 1:**
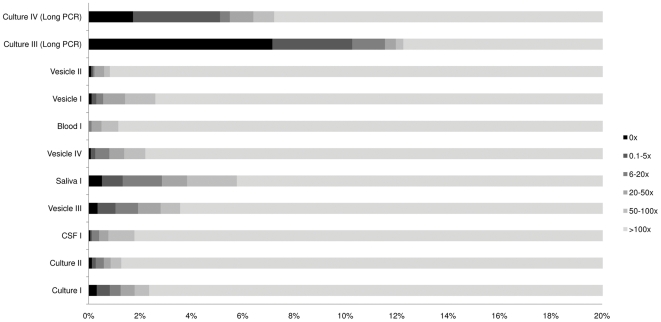
Coverage across sequenced genomes is highest using the target enrichment methods. Proportions of assembled genomes at which read depth per base falls below 100 fold (lightest grey), 50 fold, 20 fold, 5 fold, 1 fold and 0 (indicated by increasing darkness).

BLASTn [27] searches of unmapped read-pairs showed them to of human or bacterial origin with minimal homology (<30% identity) to the target enrichment probes, their presence attributed to cross-hybridisation and insufficiently stringent post-hybridisation washes. For samples prepared using the SureSelect system, 34–99% of read-pairs mapped to the reference genomes enabling the generation of full genome consensus sequences (Figure 1 and Table S1). No correlation was observed between viral load and the proportion of mapped reads. Several known short repetitive sequences within the VZV, KSHV and EBV genomes could not be accurately assembled with the BWA algorithm and are not considered further. Genome coverage was lower for samples prepared by long PCR than for target enriched sample. At mapping depths of > 5x per nucleotide, genome coverage was 94–98% for long PCR-prepared samples, compared with > 99% for target enriched samples. At mapping depths of >100x per nucleotide, genome coverage reduced to 88–92% for long PCR samples and ≥ 94% for target enriched samples (Figure S1). These differences are due to the presence of PCR-refractory regions within the VZV genome which have no effect upon the target separation and enrichment method. The specificity of the target enrichment probe sets was confirmed by our ability to specifically target and isolate either KSHV or EBV from a Primary Effusion cell line lysate infected with both viruses using independent RNA bait sets (Table 1). The successful enrichment of viral DNA in each sample is shown by the significant increase in the ratio of viral:human DNA post-hybridisation and is further evidenced by the high proportion of sequence reads that map to the target genome (Table 1).

**Figure 2:**
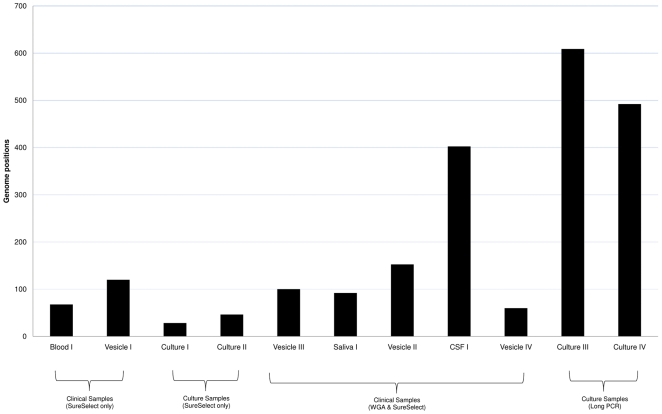
Total numbers of minority variant positions in all sequenced VZV samples. Each bar indicates the number of genome positions at which multiple alleles are present (minor allele frequency 5–49.9%). Datasets are normalised (corrected for the total number of mapped reads per sample) and showed no evidence that minority reads map to specific regions of the genome or that any bias between the proportions occurring in coding and non–coding regions of the genomes is present. Viral genome copies, post-target enrichment could not be determined for some samples (nd).

**Figure 3:**
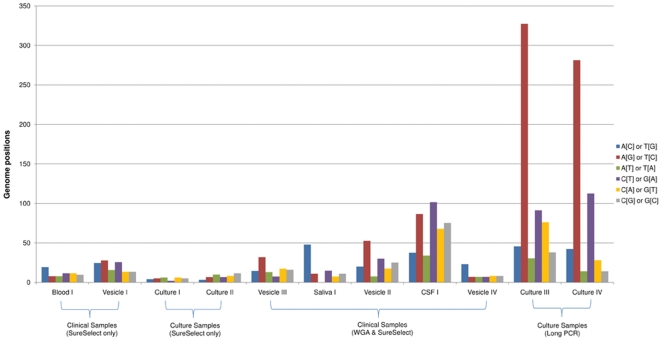
Mutational spectra of minority variants occurring within clinical samples. Each bar indicates the number of genome positions at which specific allele combinations (see graphic) are present (minor allele frequency 1–10%). Datasets are normalised (corrected for the total number of mapped reads per sample) and show a clear bias toward A to G and T to C substitutions in samples prepared by long PCR. No bias was observed in samples prepared using target enrichment methods.

Minority viral variants have been shown to be important in RNA viruses and there is evidence that diverse population structures among these viruses are strongly associated with viral evolution, disease progression and treatment failure [28], [29]. While large DNA viruses are believed to exhibit minimal genetic variation, neither the frequencies of minority variants, nor their biological importance, are known. To examine this in VZV (one of the most stable of the human herpesviruses), we defined polymorphic loci as positions at which a minor allele was present at a frequency between 5–50%, the total read depth exceeded 100 fold and a minimum of 5 independent reads carry the minor allele (Figure 2). By plotting the frequencies of each minority allele, relative to the consensus allele, we generated a ‘mutational spectrum’ for each sample showing that polymorphic loci exist at between ∼0.03–0.5% of positions in the genome (Figure 3). The frequency of VZV genome positions with minority bases was highest in two genomes (Culture III & IV) prepared by long PCR and these also showed strong bias towards A to G and T to C substitutions at minority variant positions, consistent with sequence errors introduced by *Taq*–like polymerases [30]. In contrast, no mutational pattern emerged in any samples prepared by target enrichment confirming that no systemic bias was present. For target enriched samples, those that underwent culture (Culture I and II) had the lowest numbers of minority variant positions (∼ 40–50) while the clinical samples were more variable. This likely reflects a generalised tissue culture-related loss of diversity in culture samples [8] while the relatively large proportion of polymorphic loci in CSF I may be indicative of a more diverse population structure, the significance of which is currently unknown.

These data demonstrate, for the first time, the suitability of target capture technology for purifying very low quantities of viral nucleic acid from complex DNA populations where the host genome is in vast excess. This enables deep sequencing and accurate alignment of full length viral genomes directly from clinical samples using next generation technologies, making it far superior to the culture and PCR–based methodologies. The method is sample sparing (compared to traditional techniques), compatible with WGA methods, automatable and applicable to a range of other virus genome types, including RNA viruses. We predict that the method is fully extendable to other pathogens including bacteria and protozoa present in both clinical and environmental samples. Moreover, the ability to recover multiple viral genomes from a single clinical sample using pools of different virus family capture probes offers the potential for next generation multiplex genome sequence based diagnostic testing and studies of host pathogen interactions.

## Materials and Methods

### Ethics statement

Clinical specimens (diagnostic samples collected as part of standard clinical procedures) were independently obtained from patients with confirmed VZV infection and anonymised prior to this study. Written consent was obtained in all cases. The use of these specimens for research was approved by the East London and City Health Authority Research Ethics Committee (P/96/046: Molecular typing of cases of varicella zoster virus).

### Repository of sequence read datasets

All VZV sequence datasets are available in the Sequence Read Archive under the accession number SRA030888.1. All EBV and KSHV datasets are available in the European Genome Archive under the accession EGAS00001000141.

### Sample preparation: VZV culture samples

VZV strains Culture I, II, III and IV were retrieved from the Breuer Lab Biobank and cultured (2 passages) in Mewo cells (MEM, 10% FCS, 1% Non-essential amino acids) at 34°C, 5% CO2 until 70–80% cytopathic effect was observed. The monolayer was scraped and centrifuged at 20 0g for 5 min and DNA was extracted using a QiaAmp DNA mini kit (Qiagen) according to manufacturer's instructions.

### Sample preparation: VZV diagnostic samples

Diagnostic samples from patients with confirmed VZV infection were retrieved from the Breuer lab cryobank and included vesicle fluid (Vesicle I, II, III and IV), Cerebro-spinal fluid (CSF I) and saliva (Saliva I) and 2 samples adapted to culture (Culture I & II).

Total DNA was isolated from vesicle fluid, saliva and CSF using a QiaAMP DNA mini kit according to manufacturer's instructions. Peripheral blood mononuclear cells (PMBCs) were purified from whole blood samples by centrifugation (1600 g, 15 minutes) enabling separation of plasma (top layer) and PBMCs (middle layer) from red blood cells (bottom layer) and total DNA extracted using a QIAamp DNA Blood Mini Kit according to manufacturer's instructions. Total DNA quantities were determined by NanoDrop and those with a 260/280 ratio outside the range 1.9–2.1 were further purified using the Zymoclean Genomic DNA Clean & Concentrator™ (Zymo Research Corp.).

### Sample preparation: Primary effusion lymphoma cell lines

PEL cell lines JSC-1 [31] and HBL6 [32] were cultured in RPMI containing 10% FCS (Biosera) and pen/strep (100 units ml^−1^ penicillin and 100 µg ml^−1^ streptomycin, Invitrogen). Lytic reactivation of KSHV and EBV in PEL was induced by addition of valproic acid (2.5 mg µl^−1^) and 20 ml virus-containing supernatant collected and 0.45 µm filtered after 72 hours. Viruses were concentrated using 8% Poly(ethylene glycol) triphenylphosphine (Sigma) and 0.15M NaCl. Samples were stored at 4°C for 12 hours before centrifuging (4°C, 2000 g for 10 min). The supernatant was removed and discarded and the virus pellet re-suspended into 200 µl PBS and DNA extracted using the QiaAmp DNA Blood Mini Kit (Qiagen) according to manufacturer's instructions.

### Whole genome amplification

5 clinical samples with very low total DNA quantities (with variable viral loads) were amplified (10ng starting DNA) using Genomiphi V2 (GE Healthcare) and purified using Zymoclean Genomic DNA Clean & Concentrator™ (Zymo Research Corp.), both according to manufacturer's instructions.

### Viral load assays

The relative proportions of human and viral DNA within each sample were determined by qPCR assays targeted at human GTPase KRas (KRAS) and varicella-zoster virus ORF 29.

VZV DNA was measured by a real-time PCR assay used to quantitatively detect viral DNA in clinical specimens. The PCR targets a 78 bp region in ORF 29 of the VZV genome, a 78 bp region in the EBV nuclear antigen leader protein and a 88 bp region in KSHV ORF 73. For VZV, 1 µl of sample DNA was diluted with 8 µl nuclease-free water and mixed with 12.5 µl of Qiagen master mix (from Quantitect Multiplex PCR Kit (Qiagen)), 0.94 µl (final concentration 0.94 µM) of the forward primer 5′ CACGTATTTTCAGTCCTCTTCAAGTG 3′, 0.94 µl of the reverse primer 5′ TTAGACGTGGAGTTGACATCGTTT 3′ and 0.1 µl of the FAM probe 5′ FAM- TACCGCCCGTGGAGCGCG -BHQ1 3′ (final concentration 0.4 µM). For EBV, the EBNA-LP gene was targeted and samples were prepared with the SensiMix dU kit (Bioline) using a 5 mM MgCl2 concentration, forward and reverse primers at a 20 pmolar final concentration (forward primer 5′ GGCCAGAGGTAAGTGGACTTTAAT 3′, reverse primer 5′ GGGGACCCTGAGACGGG 3′) and a probe at a 10 pmol final concentration (5′ FAM-CCCAACACTCCACCACACCCAGGC-BHQ1 3′). For KSHV, ORF 73 was targeted and samples were prepared as for EBV using the following primers and probe (Forward primer: 5′ TTGCCACCCACGCAGTCT 3′, Reverse primer: 5′ GGACGCATAGGTGTTGAAGAGTCT 3′, Probe: 5′ FAM-TCTTCTCAAAGGCCACCGCTTTCAAGTC-TAMRA 3′) [33]. Quantitative PCR was performed in a 96 well plate on an ABI 7300 or a Masterplex thermocycler ep (Eppendorf) with an initial 15 minute incubation at 95°C followed by 45 cycles at 95°C for 15 seconds and 60°C for 60 seconds. Ct values were compared to a standard curve generated using a plasmid target to assign a copy number per microliter. For human DNA, GTPase KRas was targeted using forward (5′ GCCTGCTGAAAATGACTGAATATAAAC 3′) and reverse (5′ TGATTCTGAATTAGCTGTATCGTCAAG 3′) primers at a 20pmolar final concentration. The relative proportion of human and viral DNA copy numbers was subsequently calculated and expressed as a ratio (Table 1).

### SureSelect Target Enrichment: RNA bait design

Overlapping 120-mer RNA baits (generating a 2x coverage for VZV and 5x coverage for EBV and KSHV) spanning the length of the positive strand of the reference genomes were designed using in house Perl scripts for VZV and Agilent eArray software for KSHV and EBV. For VZV, a further 552 control baits were designed against a 16 kbp region of the *Salmo trutta trutta* mitochondrion (NC_010007). The specificity of all baits was verified by BLASTn searches against the Human Genomic + Transcript database. Bait libraries for EBV, KSHV and VZV were uploaded to E-array and synthesised by Agilent Biotechnologies. All bait designs are available from the corresponding author.

### SureSelect Target Enrichment: Library preparation, hybridisation and enrichment

DNA preparations of 3 µg, 500 ng and 250 ng (the latter bulked with 2750 ng carrier DNA from MeWo cells) were sheared for 6×60 seconds using a Covaris E210 (duty cycle 10%, intensity 5 and 200 cycles per burst using frequency sweeping). End repair, non-templated addition of 3′-A, adaptor ligation, hybridisation, enrichment PCR and all post- reaction cleanup steps were performed according to the SureSelect Illumina Paired-End Sequencing Library protocol (Version 1.0) observing all recommended quality control steps.

### Long PCR

Amplicons ranging from 1–6 kbp in size and spanning the whole VZV genome were generated for culture strains 79A and V110A. 30 overlapping primer pairs were designed against the Dumas reference genome (NC_001348) as a template (Table S3). All reactions were performed using the LongAmp® *Taq* PCR Kit (NEB) and all PCR products size selected by gel purification with the QIAquick Gel Extraction Kit (Qiagen) on 0.8% 1X TAE gels stained with ethidium bromide. Cycling conditions were as follows: Denaturation at 94°C for 3 min, followed by 45 cycles of amplification (denaturation 94°C, 10 s; annealing 55°C, 40 s; extension 65°C, 30 s – 5 m) and a final extension step at 65°C for 10 min. Gel purified amplicons were merged in equimolar ratios prior to library preparation. Sequencing libraries were subsequently generated using the Nextera Tagmentation system (Epicentre Biotechnologies). Here, 50 ng of each sample was sheared and library prepped for paired end sequencing (2×54 bp) in a single reaction according to the manufacturer's instructions. Samples were tagged using the Nextera Barcode Kit and multiplexed prior to flow cell preparation and cluster generation.

### Illumina sequencing

Sample multiplexing (2 – 7 samples per lane on an 8 lane flow cell) cluster generation and sequencing was conducted using an Illumina Genome Analyzer IIx (Illumina Inc.) at UCL Genomics (UCL, London, UK) or Wellcome Trust Sanger Institute (Hinxton, UK). Base calling and sample demultiplexing were performed using the standard Illumina pipeline (CASAVA 1.7) producing paired FASTQ files for each sample.

### Sequence data processing and alignment against reference genomes

For each data set, all read-pairs were subject to quality control using the QUASR pipeline (http://sourceforge.net/projects/quasr/) to first trim the 3′ end of reads (to ensure the median Phred quality score of the last 15 bases exceeded 30) and subsequently to remove read-pairs if either read had a median Phred quality score below 30 or were less than 50 bp in length. Duplicate read-pairs were also removed. All remaining read-pairs were mapped to the reference genome using the Burrows-Wheeler Aligner (maximum insert 50 bases, maximum distance between paired ends 500) [34] generating SAM files containing all mapped and unmapped reads. SAM files were subsequently processed using SAMTools [35] to produce pileup files for consensus sequence generation and SNP calling using VarScan v2.2.3 (--min-coverage 3, --min-reads2 3, --p-value 5e-02) [23]. Unmapped read-pairs were extracted from SAM files and BLASTn searches used to determine the proportion mapping to the reference genome [27]. Read-pairs with no significant hits were subsequently checked against the non-redundant database at NCBI to determine their origin.

## Supporting Information

Figure S1 **Mean read depth across assembles genomes.** The mean read depth of each position in the assembled genome is shown for (a) VZV culture samples, (b) VZV clinical samples prepared without WGA, (c) VZV clinical samples prepared with WGA, (d) VZV long PCR samples, (e) EBV and KSHV from JSC1 cell lines and (f) EBV and KSHV rom HBL6 cell lines.
(TIF)

Table S1 **Deep sequencing of clinical samples prepared using the SureSelect Target Enrichment System.**
(DOCX)

Table S2 **Confirmation of fixed SNPs identified in assembled consensus sequences.**
(DOCX)

Table S3 **Primers used to generate overlapping amplicons by long PCR for deep–sequencing of VZV.**
(DOCX)
