# Correction: Specific Capture and Whole-Genome Sequencing of Viruses from Clinical Samples

**DOI:** 10.1371/annotation/3f1444bc-ab9d-4112-958a-2e068792f26f

**Published:** 2012-01-24

**Authors:** Daniel P. Depledge, Anne L. Palser, Simon J. Watson, Imogen Yi-Chun Lai, Eleanor R. Gray, Paul Grant, Ravinder K. Kanda, Emily Leproust, Paul Kellam, Judith Breuer

There was an error in the accession number in Materials and Methods.

EBV and KSHV dataset can be found under the Study Accession number: ERP001026.

Individual Sample Accessions are as follows:

1208_JSC1_SN_KS - ERS074823

1212_HBL6_SN_KS - ERS074824

1210_JSC1_SN_EB - ERS074825

1215_HBL6_SN_EB - ERS074833 

