# Supplementary figures and images for: Specific Capture and Whole-Genome Sequencing of Viruses from Clinical Samples
Source: PLoS One. 2011 Nov 18;6(11):e27805. doi: 10.1371/journal.pone.0027805 (PMC3220689; doi:10.1371/journal.pone.0027805)

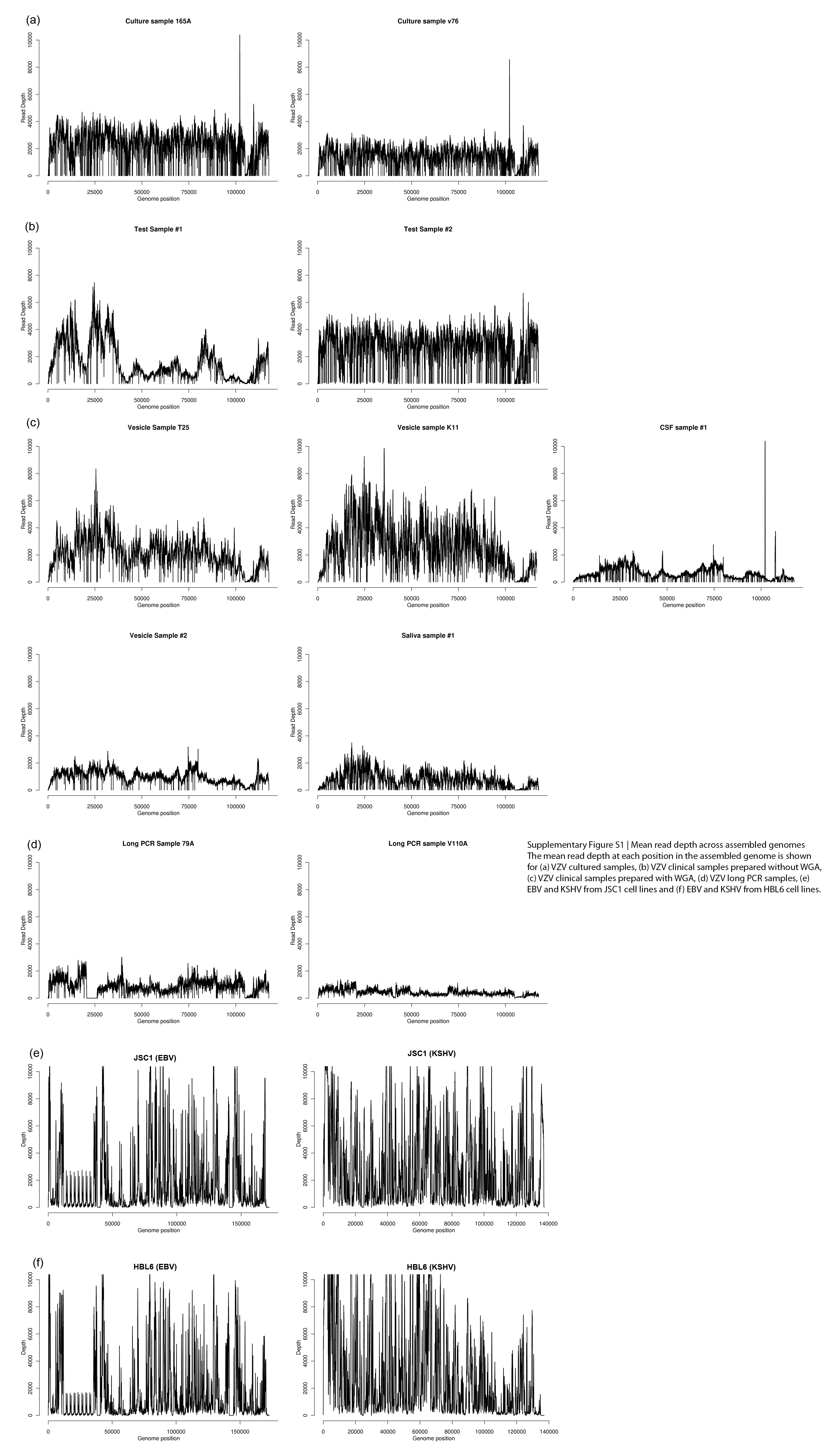

Supplement: Figure S1 — Mean read depth across assembles genomes. The mean read depth of each position in the assembled genome is shown for (a) VZV culture samples, (b) VZV clinical samples prepared without WGA, (c) VZV clinical samples prepared with WGA, (d) VZV long PCR samples, (e) EBV and KSHV from JSC1 cell lines and (f) EBV and KSHV rom HBL6 cell lines. (TIF) [file pone.0027805.s001.tif]
