# Supplementary material for: Specific Capture and Whole-Genome Sequencing of Viruses from Clinical Samples
Source: PLoS One. 2011 Nov 18;6(11):e27805. doi: 10.1371/journal.pone.0027805 (PMC3220689; doi:10.1371/journal.pone.0027805)
Supplement: Table S1 — Deep sequencing of clinical samples prepared using the SureSelect Target Enrichment System. (DOCX) [file pone.0027805.s002.docx]

|  | **Sample  (SRA ID)** | **Manipulation** | **Pre-SureSelect  viral genome**  **copies** | **Paired-end reads** | | | | **Reference**  **genome** | **% Genome coverage** | | **Mean read depth per base** |
| --- | --- | --- | --- | --- | --- | --- | --- | --- | --- | --- | --- |
|  |  |  |  | **Total** | **QC-passed** | **mapped** | **%** |  | **>5-fold** | **>100-fold** |  |
| **VZV** | Culture I  (Strain v76) | low passage culture | 2.4E+07 | 4,800,000 | 2,314,122 | 1,820,330 | 78.66 | AB097932 | 99.81 | 98.27 | 1672 |
|  | Culture II  (Strain 165A) | low passage culture | 3.2E+07 | 4,800,000 | 3,155,263 | 2,965,466 | 93.98 | AB097932 | 99.85 | 98.85 | 2720 |
|  | CSF I | WGA | 9.9E+03 | 7,200,000 | 2,290,826 | 798,874 | 34.87 | NC_001348 | 99.94 | 98.28 | 729 |
|  | Vesicle IV  (K11) | WGA | 2.0E+10 | 7,200,000 | 2,782,707 | 2,607,076 | 93.69 | AB097932 | 99.30 | 97.54 | 3022 |
|  | Saliva I | WGA | 3.5E+06 | 4,800,000 | 2,028,116 | 814,319 | 40.15 | NC_001348 | 99.19 | 94.72 | 950 |
|  | Vesicle III  (T25) | WGA | 1.9E+10 | 7,200,000 | 3,418,158 | 2,067,088 | 60.47 | AB097932 | 99.83 | 97.88 | 2416 |
|  | Vesicle III  (Ves 2) | WGA | 1.3E+05 | 7,200,000 | 1,250,864 | 1,200,918 | 96.01 | NC_001348 | 100.00 | 98.84 | 1096 |
|  | Blood I  (Test sample #1) | none | nd | 4,800,000 | 2,185,426 | 1,554,636 | 71.14 | NC_001348 | 99.82 | 97.51 | 1819 |
|  | Vesicle I  (Test sample #2) | none | 6.3E+09 | 4,800,000 | 2,719,760 | 2,705,678 | 99.48 | NC_001348 | 99.93 | 99.27 | 3197 |
| **EBV** | JSC1 | culture supernatant | 3.0E+08 | 3209053 | 2078981 | 1,436,576 | 69.10 | NC_007605 | 99.34 | 98.56 | 2523 |
|  | HBL6 | culture supernatant | 9.6E+07 | 2726837 | 1764510 | 932,367 | 52.84 | NC_009334 | 98.25 | 97.17 | 2599 |
| **KSHV** | JSC1 | culture supernatant | 7.4E+07 | 3015920 | 2074589 | 1,908,829 | 92.01 | NC_009333 | 99.73 | 95.47 | 2471 |
|  | HBL6 | culture supernatant | 5.0E+07 | 3485847 | 2358220 | 2,145,273 | 90.97 | NC_003409 | 98.19 | 93.92 | 1773 |

** below detection threshold*

**Supplementary Table S1 | Deep sequencing of clinical samples prepared using the SureSelect Target Enrichment System**
