# Supplementary material for: Specific Capture and Whole-Genome Sequencing of Viruses from Clinical Samples
Source: PLoS One. 2011 Nov 18;6(11):e27805. doi: 10.1371/journal.pone.0027805 (PMC3220689; doi:10.1371/journal.pone.0027805)
Supplement: Table S2 — Confirmation of fixed SNPs identified in assembled consensus sequences. (DOCX) [file pone.0027805.s003.docx]

| **Sample** | **Total SNPs identified** | **SNPs verified/SNPs tested** | **Methods** |
| --- | --- | --- | --- |
| Culture I | 26 | 24/24 | Previously reported ^7^ |
| Culture II | 42 | 6/6 | Direct PCR & sanger sequencing |
|  |  | 30/30 | Previously reported ^7^ |
| Vesicle II | 197 | 41/41 | long PCR and 454 sequencing |
| CSF I | 35 | 23/23 | long PCR and 454 sequencing |

**Supplementary Table S2 | Confirmation of fixed SNPs identified in assembled consensus sequences**
