# Supplementary material for: Specific Capture and Whole-Genome Sequencing of Viruses from Clinical Samples
Source: PLoS One. 2011 Nov 18;6(11):e27805. doi: 10.1371/journal.pone.0027805 (PMC3220689; doi:10.1371/journal.pone.0027805)
Supplement: Table S3 — Primers used to generate overlapping amplicons by long PCR for deep–sequencing of VZV. (DOCX) [file pone.0027805.s004.docx]

| **Amplicon** | **Range*** | **Amplicon size** | **Genomic region** | **Forward primer sequence (5' - 3')** | **Reverse primer sequence (5' - 3')** |
| --- | --- | --- | --- | --- | --- |
| 1 | 1 - 5965 | 5,966 | U_L_ | AGGCCAGCCCTCTCGCG | CACAGGGACGGCGGTTGGAC |
| 2 | 5703-10765 | 5,063 | U_L_ | CCCCGCGCTCTTTTAGAGCC | CCACGAGCCGAACAGCTGGC |
| 3 | 10513-16312 | 5,819 | U_L_ | TCGTCTAACACGCATTGCAGCG | AGTCCCGGGTAGGTGTGGGC |
| 4 | 20690-26684 | 5,995 | U_L_ | CCCTCCGTGGACCTAGCCGA | CTCGGCCGCTACCCGAAAGC |
| 5 | 15951-21015 | 5,101 | U_L_ | CTGCGGGATCGGGCTTTCGG | AGGAACGCCCAGACGGGACA |
| 6 | 26192-32198 | 6,007 | U_L_ | CGCGCGTGGACAACCTCGAT | TGGAGCAAAACAGCGCCGCA |
| 7 | 31774-37345 | 5,572 | U_L_ | CCGACCCGGAAGCGGAAACC | ACGGTCGCGATGTCGGATGC |
| 8 | 37114-40040 | 2,926 | U_L_ | AGGCGCCGGTAGAGATCCGT | GTCCCAATGAACCCCGTA |
| 9 | 39060-40843 | 1,783 | U_L_ | GGTGAGGTTACGTGGGACGATG | TTAGAAACGTGAGTTGGTGCCG |
| 10 | 40843-42907 | 2,064 | U_L_ | GCCCGTACCCTCAGACCCGT | CAGTCGCCTGGCGTCCGTTT |
| 11 | 41523-43556 | 2,033 | U_L_ | AGAACCGGGCACACAAATAG | TTGTGCGAATGCCATATGTT |
| 12 | 43467-49393 | 5,946 | U_L_ | CGTGGCGCGGGCCATAAGT | AAGGACGCGGGGTTACCGGA |
| 13 | 49051-54760 | 5,710 | U_L_ | AGAGCTTCACGTGCAACCGAAT | AAGCGACACGCCACCCTTGG |
| 14 | 54192-59492 | 5,301 | U_L_ | CCTGGAAACGCCCTGGACCG | CTTGGCGCTCAGCCGCAGAT |
| 15 | 59123-64658 | 5,536 | U_L_ | ACGGGAGCGCTGCTTTCCAC | ACGTCGAGGTTTCGCGGACG |
| 16 | 64385-69658 | 5,274 | U_L_ | CAGCTGGCGCATACCCTCGC | TAGTCGGCGCGCTGCTGATG |
| 17 | 69321-74912 | 5,592 | U_L_ | CCCGTTCGCCATATACCGCAACAA | ACCCGACTCCCAGGTCCACG |
| 18 | 74576-79919 | 5,344 | U_L_ | CTGGGAGCCAACCACCACGC | AGGCTACGGGAACGGGGACC |
| 19 | 79489-84982 | 5,494 | U_L_ | CGCCTTTAAACCAGGCGCCGA | GCGGCTCCTGAGGCTGTGTG |
| 20 | 84668-90253 | 5,586 | U_L_ | TGGCACGATCGGGATTGGATAGG | TGCCCAAGCGGGGGCAGTAA |
| 21 | 90044-95581 | 5,538 | U_L_ | TTTGGCGCCCGCGTAACTAA | ACCTGCATGCCGGGGCTCTA |
| 22 | 95229-100770 | 5,542 | U_L_ | GACAACCGCCGCTCCTCTGG | CCCTACCGCGGGACATGCAC |
| 23 | 99742-104894 | 5,973 | U_L_ | TCCGTCCGTTGAGCGGGTGT | ACGCCTCTAATGGAGTAACTGTCCCA |
| 24 | 110529-116109 | 5,581 | IR / U_S_ | GCGACAGCGTCGAGTCGGTT | ACCCCATGGTGTTCGTGTGCG |
| 25 | 115103-117562 | 2,460 | U_S_ | CCGCGACACCCAAAGGGTCC | AAGGCCAGCGTAATACATGCTTTGGT |
| 26 | 117384-118364 | 980 | U_S_ / TR | CCCCCGTAAACCCCGGAACG | GTGTGGGGACCCCGTGGGTT |
| 27 | 118124-119026 | 902 | TR | GCCCTCCCCCACAAACTCGC | AGTCGAGGCATATGCGCCGC |
| 28 | 118888-120006 | 1,118 | TR | GGGACGCTGTCTGGTGCGAC | AAACATCGGCGGGGTACCGC |
| 29 | 119805-120940 | 1,138 | TR | GCGTACACCACAGACATGCGGA | ACGGGGTCATGGTGGGACGG |
| 30 | 120755-124804 | 4,050 | TR | CCCAGTGCGATGGATACGCCG | CCGCCCGCACAGACAGACAG |

**Supplementary Table S3 | Primers used to generate overlapping amplicons by long PCR for deep–sequencing of VZV**
